# An Extract of Transgenic *Senna obtusifolia* L. hairy roots with Overexpression of PgSS1 Gene in Combination with Chemotherapeutic Agent Induces Apoptosis in the Leukemia Cell Line

**DOI:** 10.3390/biom10040510

**Published:** 2020-03-27

**Authors:** Tomasz Kowalczyk, Przemysław Sitarek, Monika Toma, Laurent Picot, Marzena Wielanek, Ewa Skała, Tomasz Śliwiński

**Affiliations:** 1 Department of Molecular Biotechnology and Genetics, University of Lodz, Banacha 12/16, 90-237 Lodz, Poland; 2 Department of Biology and Pharmaceutical Botany, Medical University of Lodz, Muszynskiego 1, 90-151 Lodz, Poland; przemyslaw.sitarek@umed.lodz.pl (P.S.); ewa.skala@umed.lodz.pl (E.S.); 3 Laboratory of Medical Genetics, Faculty of Biology and Environmental Protection, University of Lodz, 90-236 Lodz, Poland; monika.toma@biol.uni.lodz.pl (M.T.); tomasz.sliwinski@biol.uni.lodz.pl (T.Ś.); 4 Faculté des Sciences et Technologies, La Rochelle Université, UMRi CNRS 7266 LIENSs, CEDEX 1, F-17042 La Rochelle, France; laurent.picot@univ-lr.fr; 5 Department of Plant Physiology and Biochemistry, Faculty of Biology and Environmental Protection, University of Lodz, Banacha 12/16, 90-237 Lodz, Poland; marzena.wielanek@biol.uni.lodz.pl

**Keywords:** *Senna obtusifolia*, transgenic hairy roots, betulinic acid, anticancer properties, NALM6 leukemia cell line

## Abstract

Many biologically-active plant-derived compounds have therapeutic or chemopreventive effects. The use of plant in vitro cultures in conjunction with modern genetic engineering techniques allows greater amounts of valuable secondary metabolites to be obtained without interfering with the natural environment. This work presents the first findings concerning the acquisition of transgenic hairy roots of *Senna obtusifolia* overexpressing the gene encoding squalene synthase 1 from *Panax ginseng* (*PgSS1*) (SOPSS hairy loot lines) involved in terpenoid biosynthesis. Our results confirm that one of PgSS1-overexpressing hairy root line extracts (SOPSS2) possess a high cytotoxic effect against a human acute lymphoblastic leukemia (NALM6) cell line. Further analysis of the cell cycle, the expression of apoptosis-related genes (*TP53*, *PUMA*, *NOXA*, *BAX*) and the observed decrease in mitochondrial membrane potential also confirmed that the SOPSS2 hairy root extract displays the highest effects; similar results were also obtained for this extract combined with doxorubicin. The high cytotoxic activity, observed both alone or in combination with doxorubicin, may be due to the higher content of betulinic acid as determined by HPLC analysis. Our results suggest synergistic effects of tested extract (betulinic acid in greater amount) with doxorubicin which may be used in the future to develop new effective strategies of cancer chemosensitization.

## 1. Introduction

Cancer is one of the leading causes of death in the world [1]. It is expected that in developed countries, the number of cases will increase significantly in the near future, which stimulates constant work on finding effective methods for its treatment [1,2]. Of these, leukemia is the common type of blood cancer in adults and children. Despite currently available modern forms of treatment for this disease, new safer and economic alternative therapies based on compounds of natural origin are constantly being sought [3]. Plants have been a valuable source of such biologically-active substances for centuries, and have played a very important role in medicine [3,4,5]. Currently, it is estimated that about 25% of the drugs used in therapy are of plant origin [6], including a significant part proportion of current chemotherapeutics, such as vincristine, vinbastine, paclitaxel and camptothecin [7]. Nowadays, thanks to the availability of new biotechnological tools, it is possible to modify plant genomes in order to increase the production of valuable secondary metabolites and identify new ones with original modes of action that could lead to new drugs with new therapeutic properties. Importantly, such solutions often bring economic benefits that allows the cost of the final medicinal product to be reduced.

One such plant producing medically-important biologically-active compounds is *Senna obtusifolia* (L.). *S. obtusifolia* also known as *Cassia obtusifolia* or “Sicklepod” belongs to the Fabaceae family. It is a common annual plant that occurs in the Americas, Africa, Asia or Australia [8], and is considered as a remedy for many ailments, including psoriasis, rheumatism, diseases of the digestive system or eyes [9]. Literature data demonstrates that its organic extracts possess strong antioxidant, hepatoprotective, antibacterial, antifungal, anti-inflammatory, anthelmintic and anticancer activities [10,11], and the plant itself is known as a source of many valuable metabolites, such as alkaloids, polyphenols and terpenoids [11]. One of the more interesting terpenoid metabolites which may possess therapeutic properties is betulinic acid: a pentacyclic triterpene formed by betulin oxidation and obtained mainly from natural sources. Its valuable biological properties have been demonstrated in numerous in vitro and in vivo studies [12].

Recently, due to adverse climate changes, environmental pollution or other factors, a great deal of attention has been focused on obtaining valuable plant compounds from in vitro systems, which serve as potent sources of valuable secondary metabolites or recombinant proteins [13,14,15,16,17]. Among the different types of cultures currently used in plant biotechnology, hairy roots are of particular interest. This type of culture is an excellent producer of many important biologically-active compounds because they show rapid growth, can synthesize a wide spectrum of valuable metabolites and are genetically stable [18]. Additionally, modern biotechnology can employ metabolic engineering strategies based around interfering in biosynthesis pathways; these act by modifying certain enzymes that catalyze specific chemical reactions, or by making changes in proteins that regulate metabolic pathways [14]. A number of examples of effective metabolic engineering strategies have been described so far [14]. For example, our previous studies show that overexpression of AtPAP1 (*Arabidopsis thaliana* the production of anthocyanin pigment 1 (PAP1) gene encoding an MYB transcription factor) increases production of phenolic compounds in hairy roots of *Leonurus sibiricus* L. with better biological activities in comparison to untransformed roots [19]. In turn Ni et al. describe the enhancement of camptothecin production in *Camptotheca acuminata* hairy roots by overexpression of Octadecanoid-derivative Responsive *Catharanthus* AP2-domain gene (*ORCA3*). In addition, it has been shown that overexpression of squalene synthase can lead to elevate biosynthesis of triterpenes in plants [20]. Taken together, these findings indicate that the application of modern biotechnology tools, including metabolic engineering, seems to be an efficient strategy for obtaining important medicinal plants with new features.

The main aim of the present work was to obtain previously unavailable *Senna obtusifolia* hairy root cultures overexpressing *Panax ginseng* squalene synthase 1 (*PgSS1*); in addition, a second aim was to perform a quantitative analysis of betulinic acid within the culture extract and evaluate its potential to induce apoptosis, with or without doxorubicin, against a human acute lymphoblastic leukemia (NALM6) cell line.

## 2. Materials and Methods

### 2.1. Plant Expression Vector Construction

**Figure 1:**
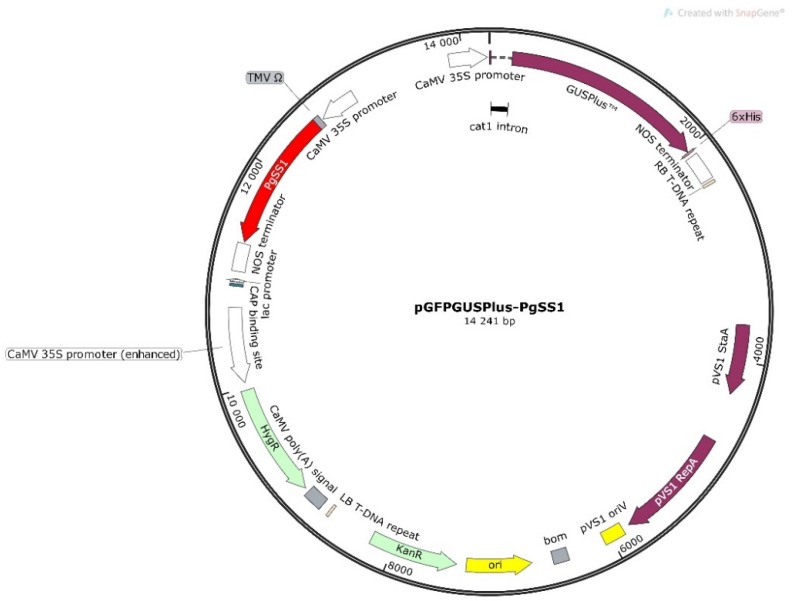
Schematic representation of the recombinant pGFPGUSPlus-*Panax ginseng* squalene synthase 1 (PgSS1) vector used in this study (SnapGene^®^ software (from GSL Biotech; available at snapgene.com)).

pGFPGUSPlus plasmid (gift from Claudia Vickers, Addgene plasmid #64401; http://n2t.net/addgene:64401; RRID:Addgene_64401) was digested by *SacI* and *XbaI* restriction enzymes to remove the enhanced green fluorescent protein (EGFP) coding sequence. The *Panax ginseng* squalene synthase 1 gene (*PgSS1*, GenBank: AB115496) was synthesized by Biomatik (Ontario, Canada) and provided in the pUC57 vector. The final version of the pGFPGUSPlus-PgSS1 (Figure 1) vector was prepared by cloning the *PgSS1* gene (containing the 5′-leader “Omega” sequence of tobacco mosaic virus (TMV) as a translational enhancer) between the constitutive Cauliflower Mosaic Virus 35S promoter (CaMV35S) and the termination sequence of the nopaline synthase gene (NOS) using *SacI* and *XbaI* restriction sites. The recombinant construct of 35S:PgSS1:NOS was verified by *SacI* and *XbaI* restriction enzyme digestion and by PCR (PgSS1: 5′-GCATGGAAGAGCTTTGACAACC-3′, PgSS2: 5′-ATGGGAAGTTTGGGGGCAAT-3′). The pGFPGUSPlus-PgSS1 recombinant vector carries also *hpt* and *gusA* genes encoding hygromycin-B-phosphotransferase (hygromycin resistance) and β-glucuronidase (reporter protein) respectively in T-DNA region (Figure 1).

### 2.2. Agrobacterium Rhizogenes Competent Cells Preparation

Competent *Agrobacterium rhizogenes* A4 cells were prepared as follows: single bacterial colonies from a YMB (yeast, mannitol broth) solid (1.5%) medium (0.065% K_2_HPO_4_ × 3H_2_O, 0.02% MgSO_4_ × 7H_2_O, 0.01% NaCl, 0.04% Yeast extract, 1% Mannitol, pH 7.5) on Petri dish were transferred into YMB liquid medium and incubated for 48 h at 28 °C on an orbital shaker at 180 rpm. Next, 100 mL of YMB liquid medium were inoculated with 2ml of bacterial culture and incubated at 28 °C on an orbital shaker at 180 rpm until the optical density (A_600_) reached 1.0. Next, the bacterial suspension was chilled on ice for 10 min and then centrifuged for 10 min at 4000 rpm. The supernatant was discarded, and the pellet was resuspended in 10 mL of sterile TE buffer (10mM Tris-HCl containing 1 mM EDTA×Na_2_) and centrifuged for 10 min at 4000 rpm. This step was repeated three times. Finally, the bacterial pellet was resuspended in 10 mL of YEM (yeast-extract-mannitol) liquid medium (0.35% Na_2_HPO_4_·12H_2_O, 0.1% K_2_HPO_4_, 0.003% MgSO_4_·7H_2_O, 0.05% NH_4_Cl, 0.4% yeast extract) and 500 µL aliquots were distributed to microfuge tubes.

### 2.3. Agrobacterium Rhizogenes Competent Cell Transformation

Aliquots of 500 µL of competent *A. rhizogenes* A4 cells were incubated on ice for 30 min and 1µg of recombinant pGFPGUSPlus-PgSS1 or pGFPGUSPlus vector was added. The tubes were incubated on ice for 15 min and then submerged in liquid nitrogen for one minute, followed by 37 °C for three minutes and then 10 min on ice. The whole bacterial suspension was then transferred to 1mL of liquid YMB medium and incubated at 28 °C for four hours on orbital shaker with gentle agitation. After this time, 100 µL of the transformed bacteria were placed on YMB agar (1.5%) plates supplemented with 50 mg/L of kanamycin and incubated for 48 h at 28 °C.

### 2.4. Plant Materials

*Senna obtusifolia* (L.) seeds were obtained from Desert Legume Program Seed Bank, The University of Arizona, Tucson, Arizona, USA.

### 2.5. Establishment of Plant In Vitro Cultures

**Figure 2:**
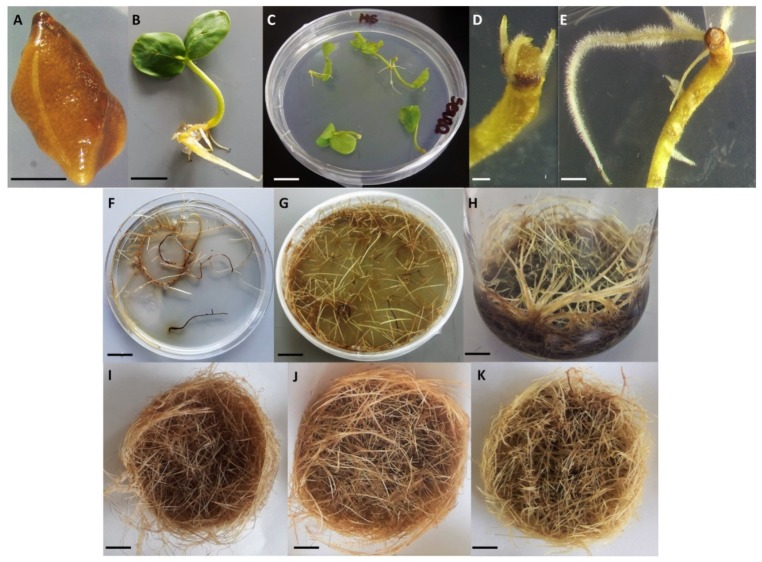
Subsequent stages of obtaining transgenic *S. obtusifolia* in vitro hairy root cultures. (**A**)- S. obtusifolia seed, (**B**)- *S. obtusifolia* 14 day old seedling used for genetic transformation, (**C**–**E**)-SOPSS hairy roots formation on *S. obtusifolia* seedlings after *Agrobacterium* transformation, (**F**)- SOPSS hairy roots after 7 days on Murashige and Skoog (MS) selection medium, (**G**)- SOPSS hairy roots after 14 days on MS solid medium, (**H**)- SOPSS hairy roots after 21 days on MS liquid medium I-K SOA4 (**I**), SOPSS (**J**), SOGFP (**K**) hairy roots after 14 days of culture on MS liquid medium used for biological studies, bar = A-2 mm, B-1 cm, C-1 cm, D-2 mm, E-5 mm, F-K-1 cm.

To establish in vitro plant cultures of *S. obtusifolia*, the seeds (Figure 2A) were surface sterilized, washed with 70% ethanol for one minute and then sterilized with 3% sodium hypochlorite containing 0.1% Triton X-100 for 10 min. Following this, the mixture was washed four times with sterile water. After the fourth wash, the seeds were left in water for three days. The sterile seeds were plated on Murashige and Skoog (MS) medium [21] containing 15 g/L sucrose and 0.8% agar. The seeds were maintained at 26 °C under 16/8 h (light/dark) photoperiod with a light intensity of 1000 lux.

### 2.6. Plant Material Transformation

*Agrobacterium rhizogenes* A4 without plasmid DNA, containing unmodified pGFPGUSPlus or pGFPGUSPlus-PgSS1 recombinant vectors, was used for plant transformation. *A. rhizogenes* harboring pGFPGUSPlus and pGFPGUSPlus-PgSS1 vector was grown on 50 mL of liquid YMB medium with 50 mg/L kanamycin at 28 °C on an orbital shaker at 180 rpm to OD_600_ = 0.7. The bacteria were then centrifuged at 4000 rpm (10 min, room temperature). The supernatant was removed and pellet diluted with the same volume of liquid MS medium supplemented with 100 µM acetosyringone.

Genetic transformation was performed using 14-day old *S. obtusifolia* seedlings (Figure 2B). For this purpose, all seedlings were cut at the middle of the hypocotyl with a surgical scalpel. The cut site of the seedling was then inoculated with a bacterial suspension and then the excess bacterium was removed on sterile paper. The inoculated seedlings were then transferred in an upside-down position (Figure 2C) on the Petri dishes with half strength MS medium and incubated at 26 °C for three days in the dark. After cocultivation plant material was rinsed in sterile water with antibiotic (cefotaxime, 250 mg/L), dried on sterile paper and transferred to Petri dishes with MS solid (1.5%) medium containing cefotaxime (250 mg/L). Incubation was carried out at 25 °C under 16h light and 8h dark. The cultures were transferred to fresh medium every seven days. The first hairy roots appeared in the cutting place on the 10th day after cocultivation (Figure 2D).

### 2.7. PgSS1, GFP and A4 Hairy Roots Selection

The well-developed hairy roots (about 2 cm) (Figure 2E) were excised from plant material and transferred on the Petri dishes with MS solid (1.5%) medium. The roots lines were transferred to MS selective medium containing 250 mg/L cefotaxime and 25 mg/L hygromycin B: this will select for the PgSS1 and GFP-expressing hairy roots, which contain the hygromycin phosphotransferase (*hpt*) gene allowing the cells to detoxify hygromycin. A4 hairy root lines were transferred on MS medium containing only 250 mg/L cefotaxime. All root lines were incubated in the dark at 25 °C. Growing hairy roots (Figure 2F) were transferred to fresh medium every 14 days. After the fourth (Figure 2G) passage, selected hairy roots were transferred to 50 mL MS liquid medium and cultured at 25 °C at 80 rpm on an orbital shaker in darkness and subcultured in MS fresh medium every 14 days (Figure 2H). The antibiotic concentration was gradually reduced (250 mg/L, 125mg/L, 62.5 mg/L, 31.25 mg/L for cefotaxime and 25mg/L, 12,5 mg/L for hygromycin B). In this experiment, we selected seven hairy root lines harboring the *PgSS1* gene (SOPSS 1-7), three hairy root lines after transformation with *A. rhizogenes* harboring the pGFPGUSPlus unmodified vector (SOGFP 1-3) and two lines after transformation with wild-type *A. rhizogenes* (SOA41-2).

### 2.8. Molecular Analysis of Hairy Roots by PCR

In order to confirm the integration of the transgene into the hairy roots genome, total genomic DNA from 7 lines (SOPSS1-7) showing the best growth rate were isolated using Genomic Mini AX Plant kit (A&A Biotechnology) following the manufacturer’s procedures. Additionally, plasmid DNA (isolated using Plasmid Miniprep DNA purification kit, EURx, Gdansk, Poland) was also isolated from *A. rhizogenes* cells as a positive control. PgSS1 sequence specific primers (For-5′-gcatggaagagctttgacaac-3′, Rev-5′-atgggaagtttgggggcaat-3′). Amplification conditions: three minutes at 95 °C; 35 cycles of 30 s at 95 °C, 30 s at 52 °C, one minute at 72 °C; finally, five minutes extension at 72 °C. Final volume of all samples was 25 μL (1 μL of each primer (10 μM), 0.5 μL of DNA (50 ng/μL), 12.5 μL of PCR Mix Plus master mix (A&A Biotechnology, Gdynia, Poland) and 11 μL of PCR Ultra-Pure Water H2O (Blirt). DNA amplification was performed in a Biometra UNO II thermal cycler. The PCR products were then visualized on 1.5% agarose gel stained with ethidium bromide.

### 2.9. β-Glucuronidase (GUSPlus) Histochemical Assay

β-glucuronidase (GUSPlus) activity in transgenic hairy roots lines was detected by histochemical test described by Jefferson et al. [22]. For this purpose, the root fragments were incubated in 5-bromo-4-chloro-3-indolyl b-D-glucuronic acid (X-Gluc) for 12 h at 37 °C. The appearance of a blue color is an indicator of GUSPlus activity.

### 2.10. GFP Expression Detection

The GFP expression of the hairy roots lines were detected under UV light with a fluorescence stereozoom microscope (SZX10, Olympus, Tokyo, Japan) and fluorescence microscope (BX60, Olympus, Tokyo, Japan). Images were analyzed with cellSens software (Olympus, Tokyo, Japan).

### 2.11. Plant Extract Preparation

Different extracts from SOA4, SOPSS and SOGFP *S. obtusifolia* hairy roots (Figure 2I–K) were included in the present study. Powdered plant materials were extracted with 500 mL of ethanol: water, 80:20 *v/v* at 35 °C using an ultrasonic bath and then twice with 300 mL of the same solvent for 15 min. The extracts were filtered off, combined and evaporated under reduced pressure, then lyophilized to dryness and left dry in the dark for further use.

### 2.12. HPLC Analysis

Identification and quantification of betulinic acid was conducted according to Pai et al. [23] with modifications. Chromatographic analysis was carried out using an HPLC system (Dionex, Sunnyvale, CA, USA) equipped with a photodiode-array detector. Chromatographic separation was achieved on a Supelcosil RP column (C18, 4.0 mm × 150 mm, 5 µm) joined with a guard column (GOLD Drop-In guards, 10 × 4 mm, 5 µm, Polygen, Gliwice, Poland) at 24 °C. Mobile phase consisted of 14% water (A) and 86% acetonitrile (B) in an isocratic mode with injection volume 20 μL. The flow rate was 1 mL/min and the absorbance was measured at 210 nm; the time of analysis was 10 min for both standards and plant extracts. Betulinic acid in plant extracts was identified by comparing the retention time and UV spectra of the analyzed samples with those of reference standards. Quantification was based on a standard curve for betulinic acid constructed over the range of 5–250 µg/mL, the linearity of the calibration curve was verified by the correlation coefficient (r^2^ = 0.9979).

### 2.13. Cell Culture

NALM6 cells from the American Type Culture Collection (Rockville, MD, USA) were cultured in RPMI 1640 (Invitrogen, Shanghai, China) supplemented with fetal calf serum (FCS; Kibbutz Beit, Haemek, Israel) and 100 units/mL penicillin and streptomycin at 37 °C in a humidified atmosphere of 95% air and 5% CO_2_.

### 2.14. Cytotoxic Activity

The cytotoxicity of SOPSS1 and SOPSS2 transgenic hairy root extracts were evaluated by assessing cell metabolic activity using the MTT assay (3-(4,5-dimethylthiazol-2-yl)-2,5-diphenyltetrazolium bromide) (Merck, Darmstadt, Germany). The procedure was performed according to the manufacturer’s instructions. Briefly, NALM6 cells were plated in 96-well plates in 100 mL of culture medium containing various concentrations (range 0-2 mg/mL) of plant extracts. Following this, 10 µL of MTT solution (final concentration 0.5 mg/mL MTT) was then added to each well and the plate was incubated in 37 °C until formazan crystals were visible. Finally, the plate was centrifuged, medium was discarded and 100 µL of solubilization solution was added into each well and the plate was allowed to stand in a humidified atmosphere of an incubator. Plates were read in a microplate spectrophotometer (Promega GloMax Plate Reader, Promega, Madison, WI, USA).

### 2.15. Gene Expression

NALM6 cells were incubated in the presence of each tested SOPSS1 and SOPSS2 transgenic hairy root extracts (alone or in combination with chemotherapeutic agent- doxorubicin 250 nM; its concentration for biological research was chosen on the basis of literature data) for 24 h. Total RNA was then extracted using an ISOLATE II RNA Mini Kit, according to the manufacturer’s instructions. cDNA was synthesized from total RNA using a High-Capacity cDNA Reverse Transcription Kit. A sample of 1 µg total RNA was used as a template in a total volume of 10 μL, following the manufacturer’s instructions. Following this, the expression of four genes (*TP53*, *PUMA*, *NOXA* and *BAX*) was analyzed by TaqMan Probe-Based Real-Time PCR Assay (Life Technologies, CA, USA), and *GAPDH* included as the reference gene. qRT-PCR was performed using TaqMan^®^ Real-Time PCR Master Mix (Life Technologies) and Agilent Technologies Stratagene Mx300SP reader (Agilent, Sant Clara, CA, USA) working on MxPro Software. The thermal cycling conditions were as follows: 10 min of polymerase activation at 95 °C, followed by 40 cycles of 30 s denaturation at 95 °C and 60 s annealing/extension at 60 °C. Each sample was run in triplicate.

### 2.16. Cell Cycle and Apoptosis

In this study, induction of apoptosis and necrosis in NALM6 was detected using an annexin V-fluorescein isothiocyanate (FITC)/propidium iodide (PI) detection kit (ThermoFisher Scientific, CA, USA) according to the manufacturer’s instructions. Briefly, cells were plated into six-well culture dishes (2 × 10^5^ cells/well) and treated with SOPSS1 and SOPSS2 transgenic hairy root extracts (alone or in combination with doxorubicin). Following 24-h incubation with both tested extracts, the percentage of apoptotic/necrotic cells was determined by the annexin V-FITC/PI assay. The content of NALM6 cells in different cell cycle phases was quantified using PI/RNase staining buffer (BD Biosciences, San Jose, CA, USA). Cells were seeded as mentioned above and treated with both extracts. After 24 h incubation, the cells were centrifuged, medium was discarded and cells were collected and fixed with 70% cold ethanol for at least one hour at −20 °C. In the next step, cells were suspended in RNase containing PI staining buffer. Cell analysis was performed using CytoFlex Flow Cytometer (Beckman Coulter, Brea, CA, USA).

### 2.17. Mitochondrial Membrane Potential (ΔΨm)

Mitochondrial membrane potential (ΔΨm) was examined using the fluorescent probe JC-1 (5,50,6,60-tetrachloro-1,10,3,30-tetraethylbenzimidazolylcarbocyanine iodide). Cells were seeded into black 96-well tissue culture plates with transparent bottom (Greiner Bio-One, Monroe, NC, USA) at a density of 1 × 105 cells/well (NALM6) in 50 µL culture medium. The cells were treated with IC_50_ of SOPSS1 and SOPSS2 transgenic hairy root extracts for 24 h (alone or in combination with doxorubicin 250 nM). Finally, the cells were pre-incubated with 5 µM JC-1 in the HBSS in a CO_2_ incubator at 37 °C for 30 min. Prior to measurements, the cells were centrifuged (300× *g* for 10 min at 22 °C) then washed twice with the HBSS. Finally, the fluorescence was measured on a Bio-Tek Synergy HT Microplate Reader according to our previous study [24].

### 2.18. Statistical Analysis

The results are expressed as mean values of three independent experiments ± SD. The Shapiro–Wilk test was used to confirm the normality of the data. The Kruskal–Wallis with multiple comparisons of mean ranks and the one-way analysis of variance (ANOVA) with the Tukey post hoc test were used to determine differences between samples. The statistical analysis was performed using STATISTICA 13.0 software (StatSoft, Poland). Differences of *p*  <  0.05 were considered statistically significant. Synergistic effect of the tested treatment was analyzed using additivity approach—two-way ANOVA.

## 3. Results

### 3.1. Generation of Transgenic Hairy Root Lines from S. obtusifolia

Hairy root lines were generated by the introduction of the *PgSS1* gene under the control of the CaMV 35S promoter (Figure 1) into *Senna obtusifolia* seedlings by *A. rhizogenes* mediated transformation. Briefly, a *PgSS1* gene (1248 bp) was used to induct seven independent clones of transgenic hairy roots (SOPSS1-7). Hairy root development was observed in the place of co-cultivation of seedlings with bacteria. The first hairy roots at the infection site appeared 10 days after the beginning of co-cultivation (Figure 2D) and the whole process of their formation was completed after 30 days. Similar induction was also observed for the hairy roots treated with type *A. rhizogenes* A4 and harboring pGFPGUSPlus vector. High transformation efficiency was observed for both the 35S::PgSS1 and 35S::GFP constructs and A4 variants (Table 1).

### 3.2. PCR analysis of Transgenic Hairy Roots

**Figure 3:**
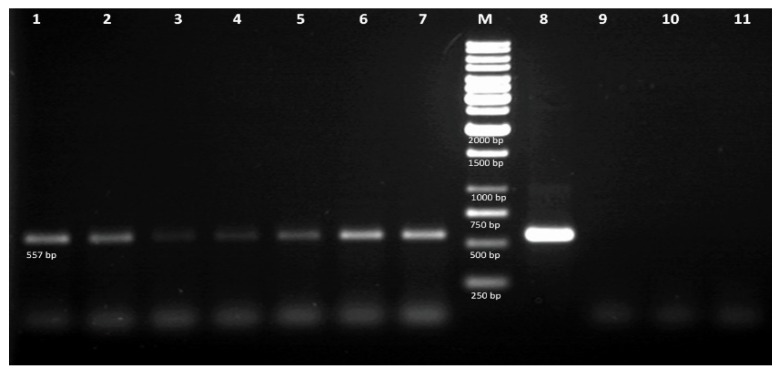
PCR analysis of transgenic hairy roots. 1-7—SOPSS 1-7 hairy root lines DNA, M—1 kb ladder, 8—pGFPGUSPlus-PgSS1 plasmid vector, 9—SOGFP hairy root line, 10—SOA4 hairy root line, 11—H_2_O.

Genomic DNA was isolated from transgenic hairy roots expressing *PgSS1*. Hairy roots with GFP expression (SOGFP) and SOA4 were used as negative control (confirmation of SOA4 root lines transformation were confirmed with *rol*B, *rol*C primers by PCR analysis-data not shown). Finally based on the results of PCR analysis, seven PCR positive lines were selected for further experiments. For SOPSS hairy root lines, the recombinant plasmid GFPGUSPlus-PgSS1 were used as positive control. The results showed the presence of bands of the expected sizes (557 bp) in all transgenic samples, in contrast to their absence in control plants (Figure 3).

### 3.3. GUSPlus and GFP Detection

**Figure 4:**
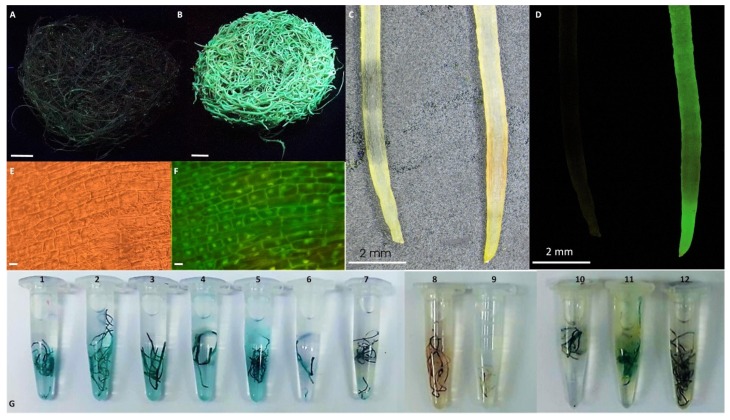
GFP fluorescence detection and histochemical analysis of β-glucuronidase activity in transgenic hairy root lines. (**A**)-SOPSS hairy roots in UV light, (**B**)- SOGFP hairy roots in UV light, (**C**)- SOPSS (left) and SOGFP (right) roots in white light, (**D**)- SOPSS (left) and SOGFP (right) roots in UV light, (**E**)-optical and (**F**)- fluorescence microscope images of SOGFP hairy root tissue, (**G**)- b-glucuronidase activity test of: 1-7—SOPSS 1-7; 8-9—SOA41,2; 10-12—SOGFP 1-3 hairy root lines. bar: A,B = 1 cm, EF = 50 µm.

To confirm the efficiency of transformation of obtained hairy roots, the β-glucuronidase gene (GUSPlus) from the pGFPGUSPlus and pGFPGUSPlus-PgSS1 vectors was used as the histochemical reporter. All higromycin B resistant lines showed positive GUSPlus expression, uniform GUS gene expression was observed along the whole root axis (Figure 4G). Analogously, green fluorescence protein (GFP) was detected for the GFP (SOGFP) control line (Figure 4A–F).

### 3.4. HPLC Analysis

**Figure 5:**
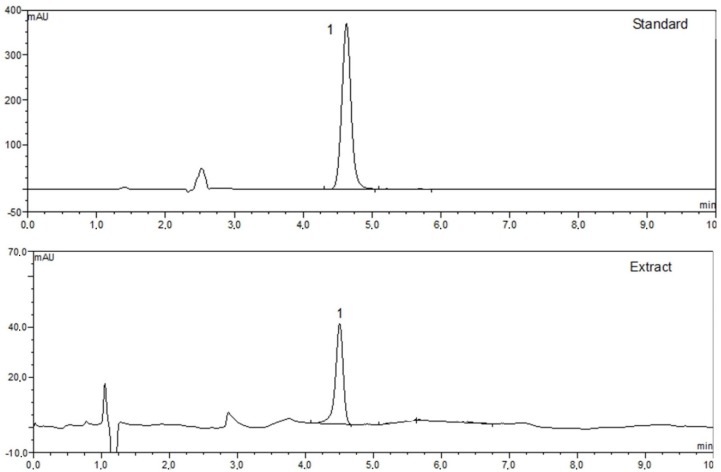
HPLC-chromatogram of SOPSS transgenic hairy root extract sample. 1 = betulinic acid.

All variants of transgenic hairy root extracts of *S. obtusifolia* were analyzed by HPLC. Our results showed that the two *A. rhizogenes*-transformed lines, SOA41 and SOA42, produced respectively, 2.779 mg/g DW and 1.948 mg/g of dry weight (DW) betulinic acid. In turn, three clones of transgenic hairy roots with GFP expression, *viz.* SOGFP1, SOGFP2 and SOGFP3, synthesized 4.088 mg/g DW, 4.7339 mg/g DW and 3.987 mg/g DW, respectively. The most productive transgenic hairy root lines were obtained by overexpression of squalene synthase (PgSS1). Betulinic acid content for the seven clones ranged between 5.998 and 22.714 mg/g DW. The highest contents of betulinic acid were measured for SOPSS1 (15.754 mg/g DW) and SOPSS2 (22.714 mg/g DW) lines, therefore these extracts were used for further biological studies (Table 2). A typical chromatogram of the aqueous methanolic extract of the *S. obtusifolia* transgenic hairy roots is shown in Figure 5.

### 3.5. Cytotoxic Activity of SOPSS1 and SOPSS2 S. obtusifolia Hairy Root Extracts in NALM6 Leukemia Cell Line

**Figure 6:**
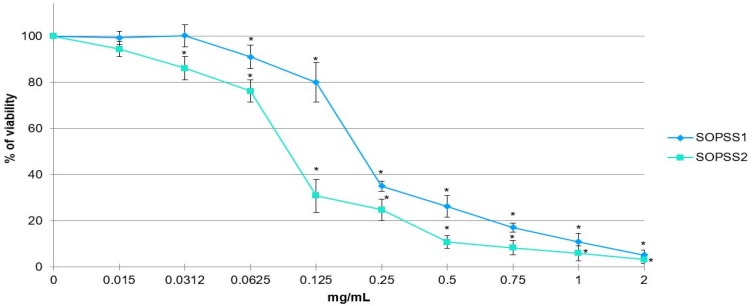
MTT assay results of transgenic SOPSS *S. obtusifolia* root extracts on human acute lymphoblastic leukemia (NALM6) cells. The cells were incubated with root extracts at a concentration range of 0.015–2 mg/mL for 24 h. Data are presented as the mean ± SD of triplicate independent experiments. * *p* < 0.05 SOPSS1, SOPSS2 vs. untreated cells.

The cytotoxicity of two transgenic hairy root extracts against NALM6 cancer cells are summarized in Figure 6. A significant decrease in cell viability noted by MTT assay, was recorded with increasing SOPSS1 and SOPSS2 root extract concentrations across the tested range (0.015–2 mg/mL). Both tested extracts were able to kill leukemia cancer cells in vitro and exerted a high cytotoxic activity toward the cell line but better activity was observed for the SOPSS2 root extract. The IC_50_ for the SOPSS1 root extract was 0.23 mg/mL; however, this amount fell to 0.08 mg/mL for the SOPSS2 root. Therefore, the IC_50_ of both tested extracts, comprised in the range 0.08 to 0.23 mg/mL was selected for further study in NALM6 cells.

### 3.6. Gene Expression Analysis

**Figure 7:**
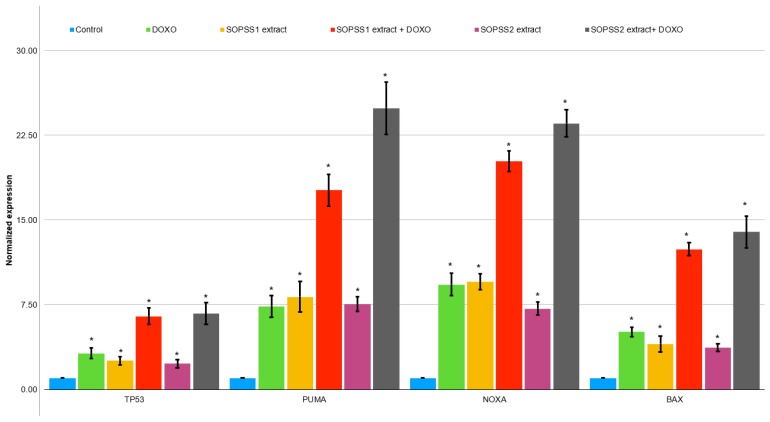
Expression analysis of apoptotic genes (*TP53*, *PUMA*, *NOXA* and *BAX*) in NALM6 cells treated with IC_50_ of SOPSS1 and SOPSS2 transgenic hairy root extracts, alone or co-treated with 250 nM doxorubicin, for 24 h. The gene expression was determined as described in RT-PCR methodology. Data presented as mean ± SD of at least three independent experiments. * *p* < 0.05 SOPPS1 or SOPSS2 alone or in combination with doxorubicin) vs. control.

The NALM6 cell line was exposed to SOPSS1 and SOPSS2 transgenic hairy root extracts at doses corresponding to IC_50_ values for 24 h. Following this, total RNA was successfully isolated from cells to study the expression of apoptosis related genes. RT-PCR assay of apoptosis-related genes (*TP53*, *PUMA*, *NOXA* and *BAX*) was tested in NALM6 cell line after exposure to both extracts alone and in combination with doxorubicin. Our studies revealed an increase in the expression level of all apoptotic genes after the treatment with the extracts alone and in combination with 250 nM doxorubicin compared to the untreated control; however, a higher effect was observed for the SOPSS2 root extract, both alone and in combination with doxorubicin. The changes in expression level of the examined genes in response to exposure to SOPSS1 and SOPSS2 transgenic hairy root extracts at IC_50_ (alone and in combination with doxorubicin) are presented in Figure 7.

### 3.7. Effects of SOPSS Transgenic Hairy Root Extracts (Alone or Combination with Doxorubicin) on Apoptosis and Cell Cycle in NALM6 Cells

**Figure 8:**
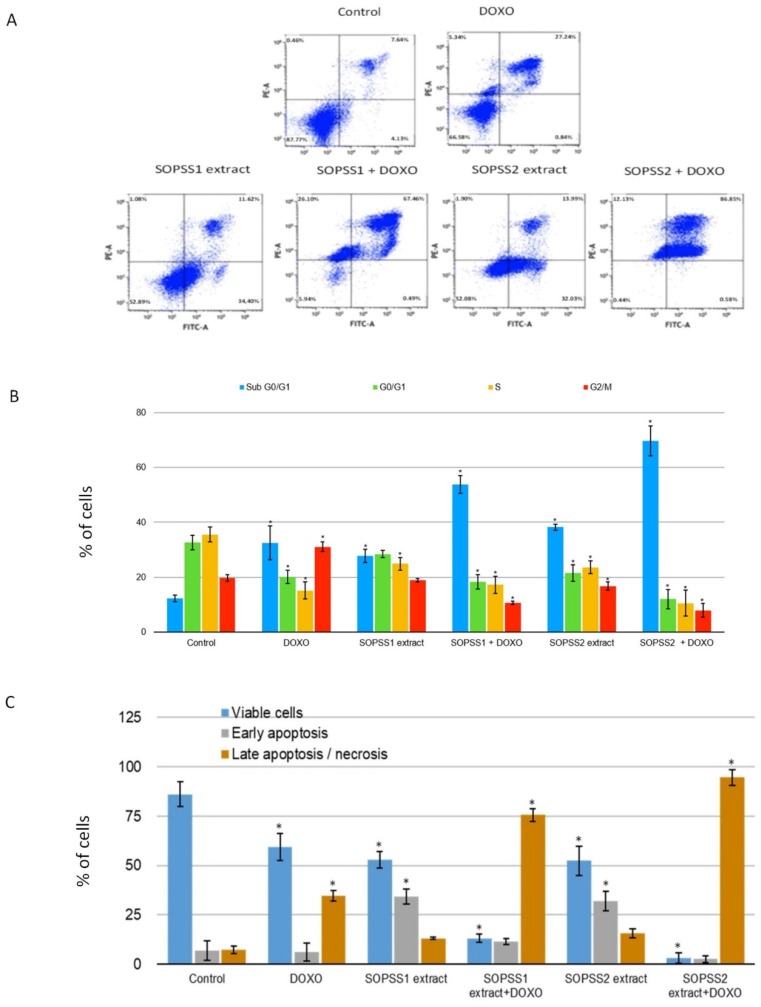
(**A**) Apoptosis induction by transgenic SOPSS1 and SOPSS2 root extracts at their IC_50_ alone or in combination with doxorubicin on NALM6 cells for 24 h determined by Annexin V-FITC/PI flow cytometry-representative cytograms. (**B**) The diagram shows the percentage of the cell population in each phase of the cell cycle in NALM6 cells after 24-h treatment with SOPSS1 and SOPSS2 root extracts (alone or combination with doxorubicin). (**C**) Diagram shows percentage of cell populations divided to viable cells, early apoptotic, late apoptotic/necrotic cells. Data represent the mean ± SD of three independent experiments. * *p* < 0.05 SOPPS1 or SOPSS2 (alone or in combination with doxorubicin) vs. control.

The effect of SOPSS1 and SOPSS2 transgenic hairy root extracts (alone or combination with doxorubicin) treatment on the cell cycle distribution and apoptosis was ascertained using flow cytometry. A greater percentage of apoptotic cells was observed following treatment with SOPSS1 or SOPSS2 at IC_50_ for 24 h compared to controls (Figure 8A). Additionally, co-treatment with the chemotherapeutic further increased this activity. More effective result was observed for the SOPSS2 root extract alone or in combination with doxorubicin (Figure 8B). These findings confirm the effectiveness of the tested extracts alone or in combination with a chemotherapeutic agent.

The role of SOPSS transgenic hairy root extracts in cell death (anticancer/cytotoxic potential) in NALM6 cells was evaluated using cell cycle analysis. Twenty-four hour exposure to either extract at IC_50_ for 24 h resulted in an increase in sub-G0/G1 cell population in the NALM6 cells as compared to untreated control; similar results were also obtained for the combination of the extract with doxorubicin. Exposure to IC_50_ SOPSS1 resulted in a 27% increase in the sub-G0/G1 phase of NALM6 cells, as well as a 39% increase for SOPSS2, clearly indicating elevated cell death; in contrast, untreated controls displayed a 12% increase. In addition, doxorubicin plus SOPSS1 was found to elicit a 53% increase and doxorubicin plus SOPSS2 extract a 70% increase.

### 3.8. Effect of Transgenic SOPSS Root Extracts (Alone or Combination with Doxorubicin) on Mitochondrial Membrane Potential of NALM6 Cells

**Figure 9:**
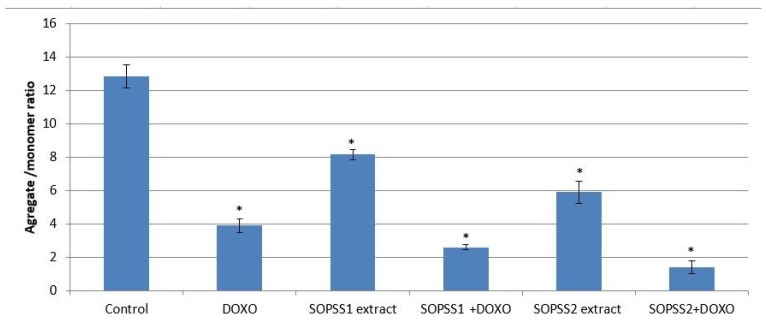
Mitochondrial membrane potential upon exposure to transgenic SOPSS1 and SOPSS2 root extracts (alone or with combination with IC_50_ doxorubicin) for 24 h on NALM6 cells. Results are shown as mean ± SD (n = 3),* *p* < 0.05 control vs. transgenic SOPSS1 and SOPSS2 hairy root extracts (alone or combination with doxorubicin). Doxorubicin-treated cells are used as a positive control.

As the disruption of mitochondrial membrane potential (MMP) plays a major role in the apoptosis mechanism, the next stage examined whether transgenic SOPSS1 and SOPSS2 hairy root extracts mediated NALM6 cell death through the intrinsic apoptosis pathway. In addition, we checked the effect of both extracts in combination with a chemotherapeutic agent. Our studies showed that transgenic SOPSS1 and SOPSS2 hairy root extracts, either alone or in combination with doxorubicin, induced apoptosis as evidenced by the loss of MMP; however, a higher effect was observed for the SOPSS2 extract, alone or in combination with doxorubicin (Figure 9).

## 4. Discussion

Transgenic plants are increasingly becoming an extremely attractive source of desirable biologically-active compounds [25,26,27,28,29,30,31]. The dynamic development of genetic engineering tools now allows plants to be modified for the most efficient production of many therapeutics, such as recombinant proteins or secondary metabolites. A wide range of pharmaceutical proteins for therapeutic use is currently in different phases of clinical trials [32]. It is also possible to produce secondary metabolites with high yields using routine techniques of in vitro plant cell and tissue cultures, which are becoming an attractive alternative to traditionally-used, naturally occurring plant material [33,34].

Interestingly, transgenic hairy roots formed after genetic transformation of plant material by *A. rhizogenes* are an ideal source of such biologically active compounds [35,36]. To date, such roots have been obtained from at least 150 plant species from 41 families. Interference with selected elements of their metabolic pathways can lead to overproduction of many valuable metabolites [37], one of which is squalene synthase (SS, EC 2.5.1.21): a key enzyme that catalyzes the first enzymatic step in the biosynthesis of triterpenoids, such as betulinic acid [38]. Such compounds have significant medicinal and economic value [39,40,41].

The aim of our work was to create a previously unobtained transgenic hairy root culture of *Senna obtusifolia* overexpressing squalene synthase from *Panax ginseng*, and to perform a molecular analysis of the integration of a transgene with the nuclear DNA of generated hairy root lines. Our results confirm the transgenic nature of agro-transformation-generated hairy root clones by PCR, and GUS positive test for *PgSS1* containing clones (SOPSS). In addition, GFP expressing lines (SOGFP) were confirmed by detection of their fluorescence in comparison to lines not containing the gene encoding the GFP protein (SOA4). The betulinic acid content in the various types of transgenic root lines was then confirmed. Finally, the biological properties of the most productive line extract (based on betulinic acid content) and their potential anti-cancer effect in combination with a chemotherapeutic agent (doxorubicin) on leukemia NALM6 cells were then determined.

Literature data show that *S. obtusifolia* hairy roots grown in vitro can be a rich source of many valuable biologically-active compounds. Ko et al. [37] obtained hairy roots of *S. obtusifolia* to study properties of five anthraquinones such as chrysophanol, emodin, physcion, 8-O-methylchrysophanol and 1,8-di-O-methylochrysophanol. In turn, Guo et al. [42] showed the presence of various anthraquinones and betulinic acid, sigmasterol and sitosterol in *S. obtusifolia* hairy roots and the ability to influence their production by changing the culture conditions. In our studies, the hairy roots obtained after transformation with *A. rhizogenes* A4 did not show significant differences in the content of betulinic acid in comparison to non-transformed roots (data not shown). Unlike Guo et al., the present study uses a new metabolic engineering strategy based around the overexpression of a key enzyme in the terpenoid biosynthesis pathway (squalene synthase) to improve betulinic acid productivity.

The present study describes the production of seven transgenic hairy roots lines of *S. obtusifolia* (SOPSS1-7); these were subjected to HPLC analysis to select the most productive lines for betulinic acid biosynthesis. This lupane-type triterpenoid, a product of betulin oxidation, possesses proven anticancer properties in many lines of human cancer cells. Betulinic acid is present in nature in many plant species such as *Betula* sp., *Vitex negundo* or *Combretum quadrangulare*. Our previous work found it to be present in *Menyanthes trifoliata* where it was found at higher levels in roots derived from in vitro cultures than in aerial parts [43].

In the first step of this work, after all hairy root lines were obtained, the level of betulinic acid in the tested extracts was determined. The highest content was found in transgenic hairy root lines (SOPSS1-7), where it ranged from 5.99 mg/g DW to 22.71 mg/g DW (Table 2). This is the first report showing an attempt to increase betulinic acid production in transgenic hairy root with constitutive overexpression of squalene synthase. Seo et al. [44] showed that transgenic *Eleutherococcus senticosus* Rupr. and Maxim. plants generated by the introduction of a *PgSS1* gene demonstrated increased production of triterpenoids after transformation with *Agrobacterium*. However, Lee et al. [45] introduced the same gene into *Panax ginseng*, resulting in the production of transgenic plants with increased triterpene biosynthesis. In turn, Mirjalili et al. [46] obtained *Withania coagulans* transgenic hairy roots in which the squalene synthase gene was under the control of the CaMV35S promoter, and its overproduction led to effective elevation of triterpenoids. These examples, together with our results, show that this metabolic engineering strategy is a great tool for increasing the level of particularly valuable metabolites in plant in vitro cultures that naturally produce relatively lower amounts.

The present study examined the cytotoxic effect of *S. obtusifolia* transgenic hairy root extracts on leukemia cells (NALM6). Our results show that the IC_50_ value for the extracts from *S. obtusifolia* hairy roots obtained in the MTT assay was 0.08 mg/mL for the SOPSS2 roots and 0.23 mg/mL for the SOPSS1 roots. Of the two, the SOPSS2 extract showed a stronger cytotoxic effect on NALM6 cells. It is possible that this stronger cytotoxic may be due to the higher content of betulinic acid in the analyzed transgenic hairy roots overexpressing *PgSS1*. Previous studies have so far addressed only the cytotoxic effect of *Senna* spp. extracts derived from wild, ex vitro, untransformed plants: Despande et al. [47] report that stem extract of *S. obtusifolia*, *S. glauca* and *S. sophera* exhibited cytotoxic effects against breast cancer cell lines, while others report cytotoxic effects following treatment with *S. nigricans* on MCF-7 (adenocarcinoma estrogen-responsive breast cancer) and *S. alata* extracts on A549 (epithelial human lung adenocarcinoma cancer) cell lines [48,49,50].

The final stage of our research identified induction of apoptosis through cell cycle arrest in the subG0/G1 phase, a decrease of mitochondrial membrane potential and a change in the expression level of selected apoptosis-related genes (*TP53*, *PUMA*, *NOXA* and *BAX*) in NALM6 cells after treatment with transgenic hairy root extracts. These were observed for treatment with *S. obtusifolia* alone and in combination with doxorubicin.

Many studies have examined the potential for plant-derived biologically-active compounds to induce apoptosis in cancer cells, with very promising results being obtained by the combination of the extract with a synthetic anti-cancer drug [51,52,53,54,55]. One such commonly-used anti-cancer agent is doxorubicin which is commonly used in the treatment of a wide range of cancers, including, different types of carcinomas or hematological malignancies. It is known to intercalate with DNA and inhibit macromolecular biosynthesis [56]; such a combination may elicit a stronger anti-cancer effect, and hence act as an effective potential therapeutic method of cancer treatment. Many currently-used anticancer drugs are based on targeting the apoptotic signaling pathway [57].

Apoptosis is known to be induced by many external and internal factors, and controlled by a number of cell signals. The intrinsic apoptosis pathway can be promoted by DNA damage caused by cytotoxic agents. Mitochondria, and more precisely the permeability of their outer membrane, play an extremely important role in this process. The permeability of the mitochondrial outer membrane allows signaling molecules to be released from the intermembrane space into the cytosol and triggers caspase activation. Such membrane permeabilization, and the consequent release of apoptogenic factors, can be initiated by well-known pro-apoptotic factors, such as p53, and the Puma, Noxa and Bax proteins of the Bcl-2 family [57].

Treatment with two tested extracts (SOPSS1 or SOPSS2) or co-treatment with IC_50_ doxorubicin significantly increased cell population in sub-G1 phase. In addition, both extracts (alone or in combination with doxorubicin) elevated the expression of apoptosis-related genes. Although both extracts induced apoptosis in leukemia cells, the overexpressing PgSS1 (SOPSS2) line in combination with doxorubicin showed a stronger effect on NALM6 cells. It is likely that the stronger pro-apoptotic activity of the SOPSS2 extract may be associated with its greater betulinic acid content. However, there is currently a lack of literature data examining the influence of extracts from transgenic hairy roots of *S. obtusifolia* on the induction of intrinsic apoptosis in cancer cells.

Some reports indicate that plant extracts containing betulinic acid may have anti-cancer properties. Soica et al. [58] analyzed the betulin and betulinic acid content of a birch tree bark extract, as well as its cytotoxic activity against skin epidermoid carcinoma (A431), breast carcinoma (MCF7) and cervix adenocarcinoma (HeLa) cell lines where high proliferation inhibition was described. Additionally, Drag et al. tested the activity of crude birch bark extract on human gastric carcinoma and human pancreatic carcinoma cell lines. Their results suggest that betulinic acid may represent a promising lead in the treatment of cancer [59].

Ehrhardt et al. found betulinic acid to induce apoptosis in NALM6 leukemia cell line by exposure of phosphatidylserine on the cell surface followed by an increase in sub-G1 peak of DNA fragmentation [60]. The same authors described that the combination of betulinic acid with doxorubicin induced p53- and NOXA-dependent cell death proceeding by mitochondrial pore formation, release of cytochrome c and caspase activation [61]. Similarly to the mechanism described by the authors, our results suggest that the induction of apoptosis in leukemia cells is influenced by the activation of apoptotic genes (*TP53*, *PUMA*, *NOXA* and *BAX*), cell cycle arrest in the subG0/G1 phase and decrease of mitochondrial membrane potential.

Interestingly, of the two tested extracts, SOPSS2 contained a higher concentration of betulinic acid and showed stronger biological properties than SOPSS1. The combination of a traditional chemotherapeutic and an extract containing betulinic acid appears to be an effective anti-cancer strategy, but further investigations are needed to better understand the molecular mechanisms. Further studies using different extract fractions or isolated pure compounds in combination with doxorubicin are planned. This strategy, combined with well-developed methods for obtaining plant in vitro cultures, may in the future lead to finding biologically active compounds that could be used in clinical research and potentially cancer therapy Many experimental data indicate that the combination of conventional drugs with biologically active plant-derived compounds (in various forms) can reduce the systemic toxicity of anticancer therapy because they allow the reduction of the concentration of toxic drug.

## 5. Conclusions

Our findings confirm that highly-productive in vitro cultures of transgenic hairy roots of *S. obtusifolia* overexpressing squalene synthase can be an easy and cheap system to produce with high yields terpenoids of relevant medical use. In addition, this approach to obtaining potential phytopharmaceuticals does not interfere with natural plant habitats and removes the dependency on naturally-occurring plant material which can be limited by pollution and climate changes. Our results demonstrate that the combination of plant extracts obtained from in vitro plant cultures containing valuable secondary metabolites, such as betulinic acid, with traditionally-used chemotherapeutics, such as doxorubicin, has a stronger cytotoxic effect on leukemia (NALM6) cells than betulinic acid alone; this combination acts by inducing apoptosis through cell cycle arrest in the subG0/G1 phase, altering the level of apoptotic-related gene expression (*TP53*, *PUMA*, *NOXA* and *BAX*) and reducing mitochondrial membrane potential. It is possible that the extracts may sensitize cancer cells to a synthetic anticancer agent, thus enhancing their therapeutic effect. Therefore, further in vivo studies are needed to evaluate the potential of such transgenic hairy root extracts containing increased amounts of betulinic acid as natural compounds for use in combination with chemotherapeutic agents such as doxorubicin.

## Figures and Tables

**Table 1:** Efficiency of hairy root formation on *Senna obtusifolia* seedlings.

Genetic Construct	Number of Seedlings Used for Transformation	Number of Seedlings with Hairy Root Induction	Hairy Root Induction Efficiency (%)
35S::PgSS1	60	48	80
35S::GFP	60	52	87
A4 hairy roots	60	55	92

**Table 2:** Content of betulinic acid (mg/g DW) in extracts from different lines of *S. obtusifolia* hairy roots.

Roots Extracts from Various Clones of *S. obtusifolia*	Betulinic Acid mg/g DW
SOA41	2.7795 ± 0.0168 ^a^
SOA42	1.9482 ± 0.0172 ^b^
SOGFP1	4.0882 ± 0.2986 ^b^
SOGFP2	4.7339 ± 0.0254 ^a^
SOGFP3	3.9875 ± 0.0238 ^b^
SOPSS1	15.7548 ± 0.4891 ^b^
SOPSS2	22.7144 ± 0.0267 ^a^
SOPSS3	8.6489 ± 0.3644 ^f^
SOPSS4	5.9987 ± 0.0457 ^g^
SOPSS5	14.4669 ± 0.0514 ^c^
SOPSS6	10.9742 ± 0.092 ^d^
SOPSS7	9.0251 ± 0.0494 ^e^

Different superscript letter within each variants indicate significant differences in the mean values at *p* < 0.05.
